# Supplementary figures and images for: Ablation of palladin in adult heart causes dilated cardiomyopathy associated with intercalated disc abnormalities
Source: eLife. 2023 Mar 16;12:e78629. doi: 10.7554/eLife.78629 (PMC10069870; doi:10.7554/eLife.78629)

Figure 2—source data 2. Uncropped Western blots for Figure 2B.

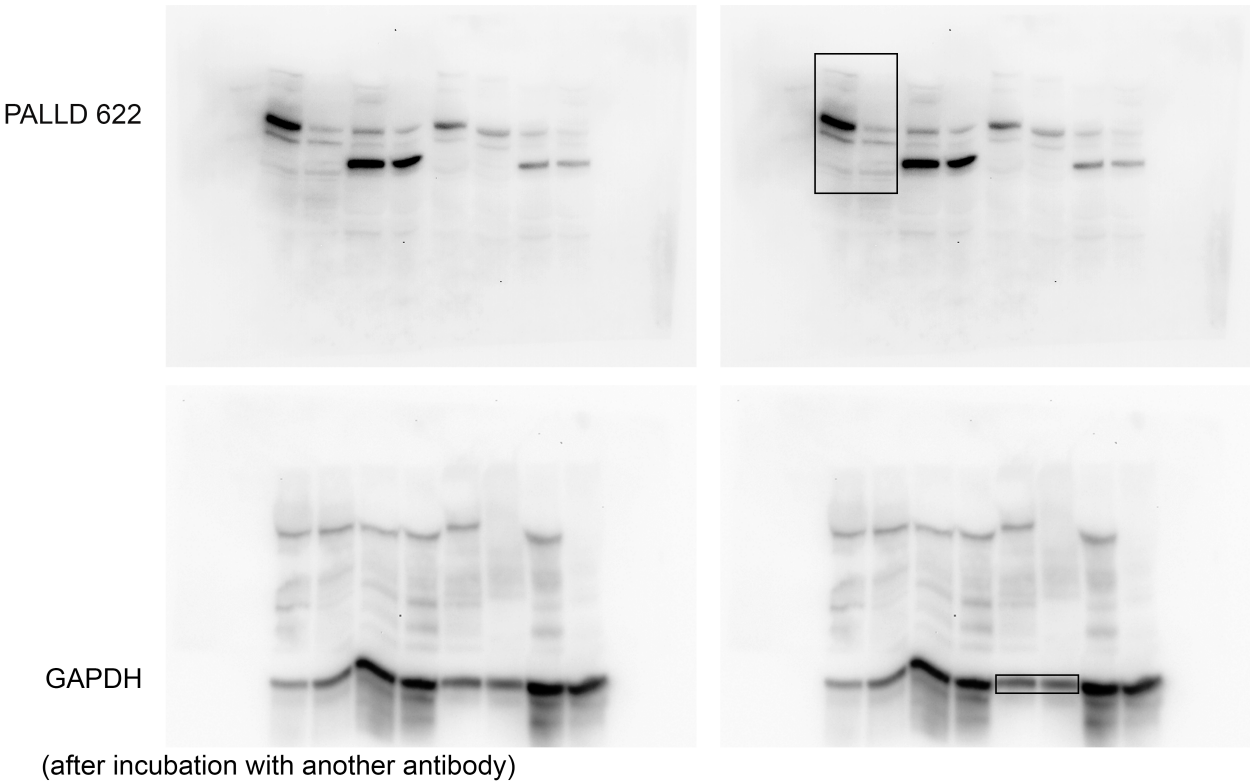

Supplement: Figure 2—source data 2. [file elife-78629-fig2-data2.pdf]

Figure 3—source data 2. Uncropped Western blots for Figure 3B.

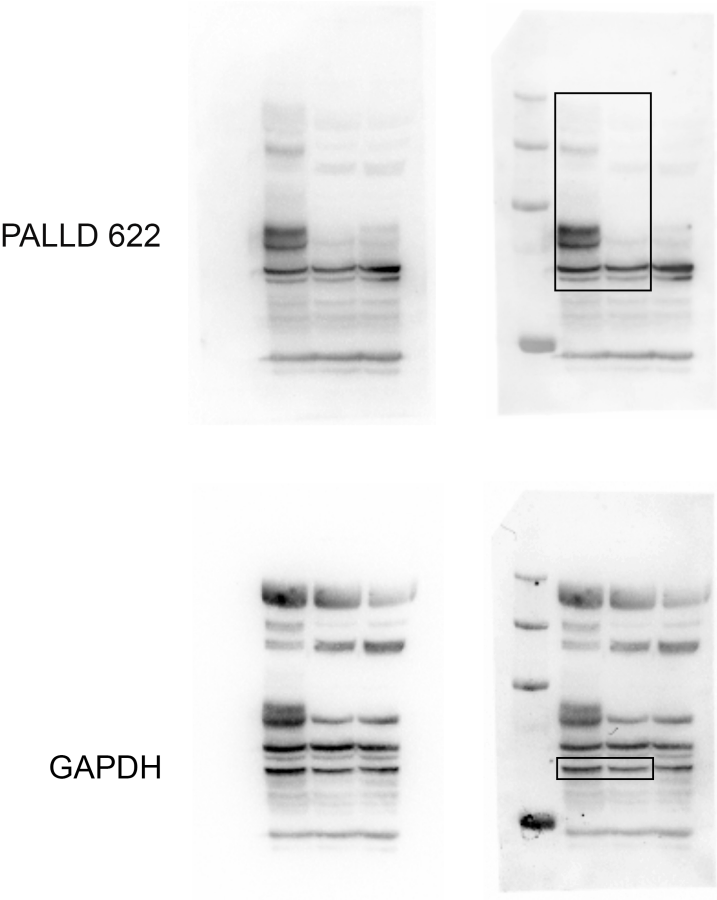

Supplement: Figure 3—source data 2. [file elife-78629-fig3-data2.pdf]

**Figure 6—source data 1. Uncropped Western blots for Figure 6A.**

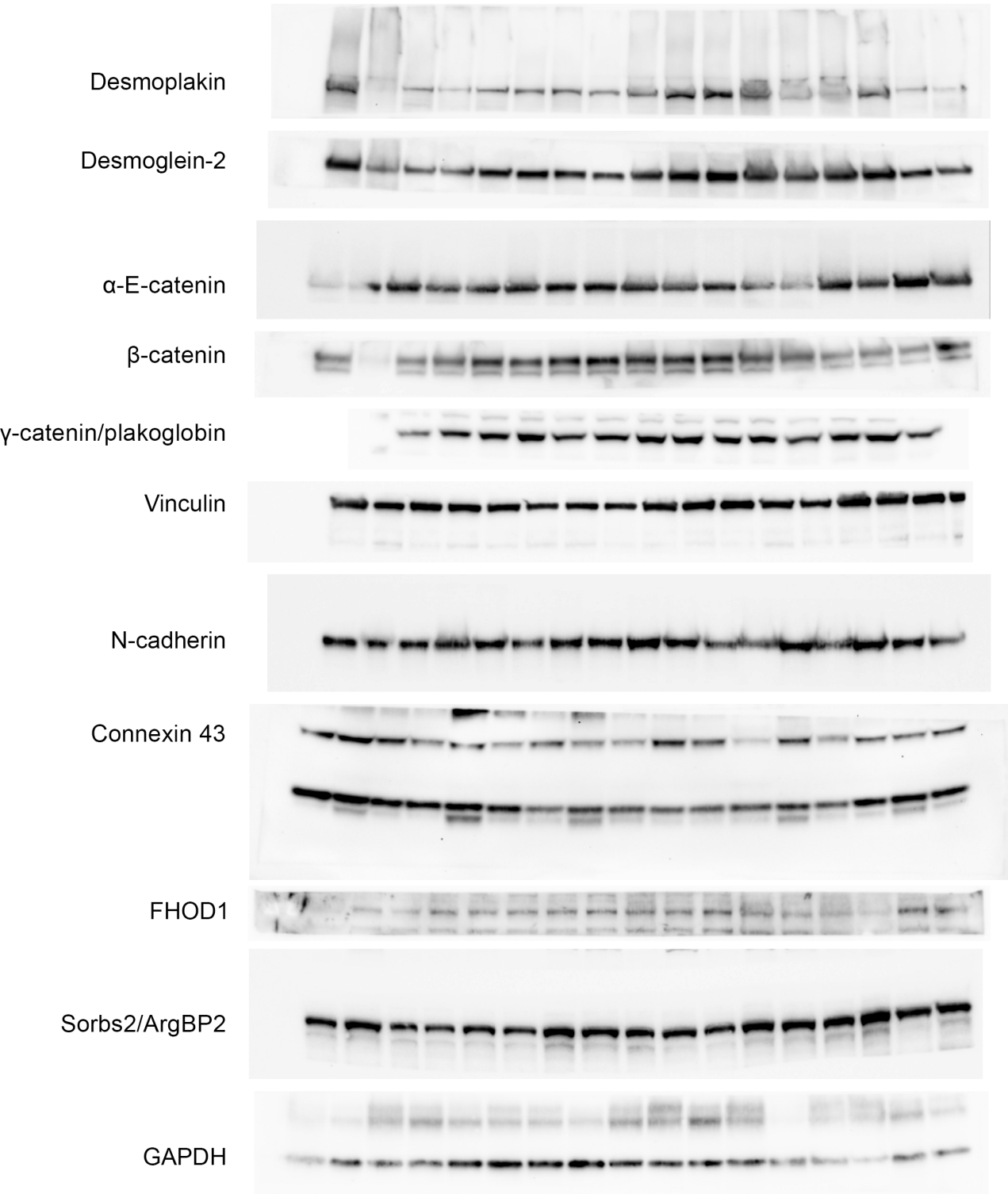

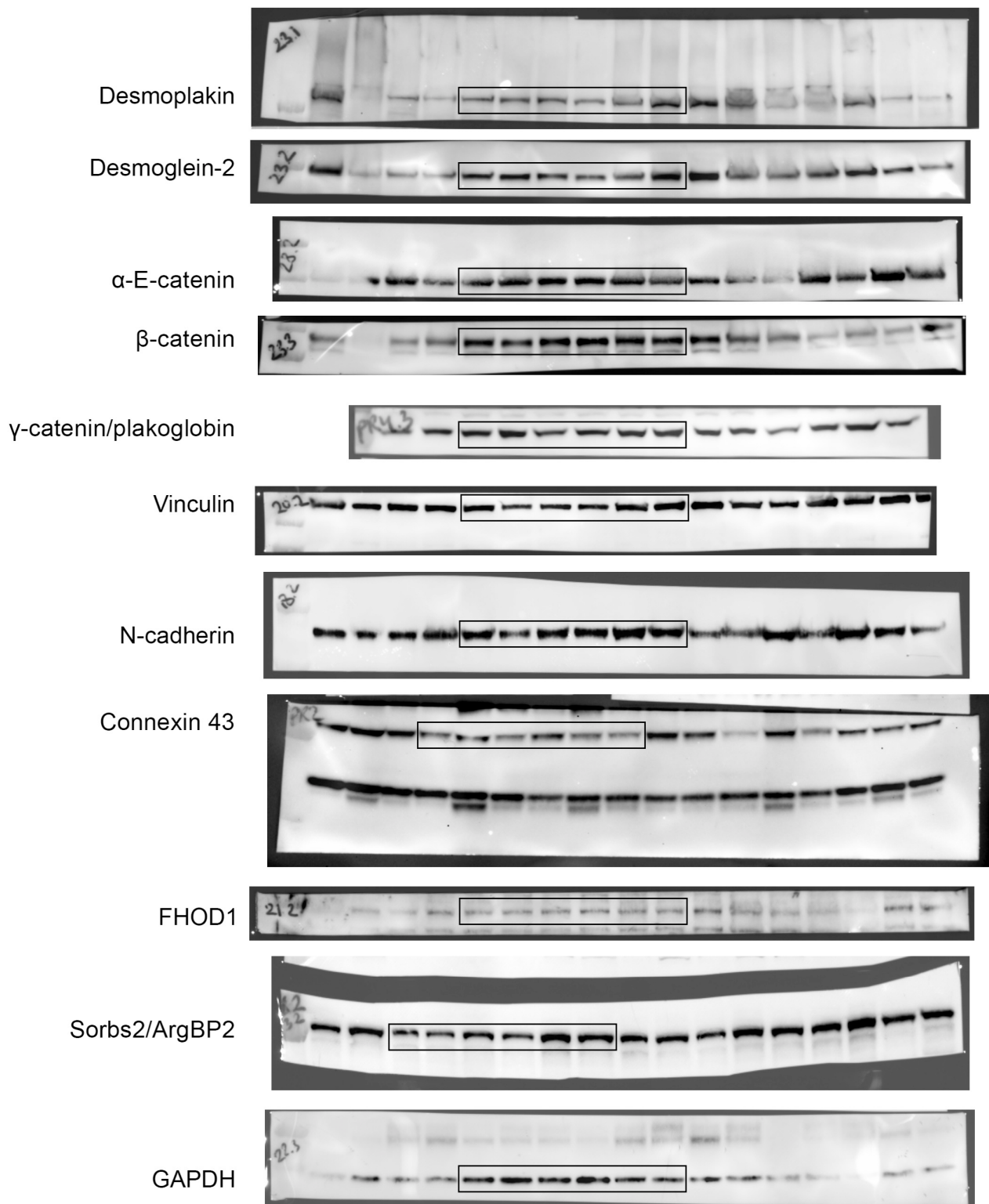

Supplement: Figure 6—source data 1. [file elife-78629-fig6-data1.pdf]

Figure 6—source data 4. Uncropped Western blots for Figure 6D.

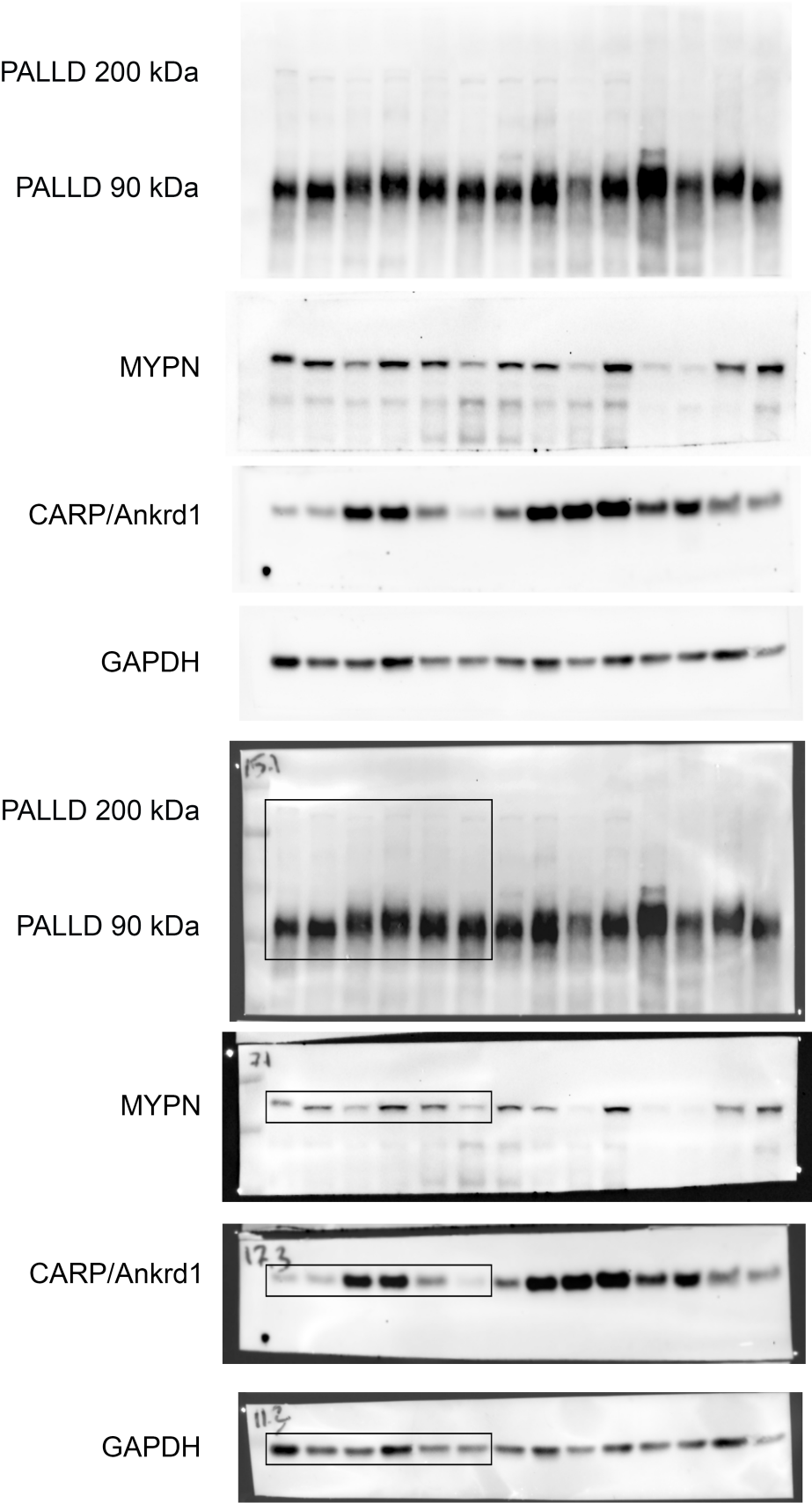

Supplement: Figure 6—source data 4. [file elife-78629-fig6-data4.pdf]
