# Supplementary material for: Ablation of palladin in adult heart causes dilated cardiomyopathy associated with intercalated disc abnormalities
Source: eLife. 2023 Mar 16;12:e78629. doi: 10.7554/eLife.78629 (PMC10069870; doi:10.7554/eLife.78629)
Supplement: Figure 2—source data 3. [file elife-78629-fig2-data3.docx]

**Figure 2–source data 3.** Echocardiographic parameters of 2-month-old male cPKO mice compared to controls before and 4 weeks after mechanical pressure overload induced by transaortic constriction (TAC).

|  | **Before TAC** | | | | **4 weeks after TAC** | | |
| --- | --- | --- | --- | --- | --- | --- | --- |
|  | ***Palld^fl/fl^***  **(*n* = 9)** | ***Myh6^Cre/+^***  **(*n* = 14)** | ***Palld^fl/fl^; Myh6^Cre/+^***  **(*n* = 13)** | ***Palld^fl/fl^***  **(*n* = 4)** | | ***Myh6^Cre/+^***  **(*n* = 9)** | ***Palld^fl/fl^; Myh6^Cre/+^***  **(*n* = 7)** |
| **Age (weeks)** | 6.9 ± 0.2 | 7.4 ± 0.3 | 7.0 ± 0.3 | 11.8 ± 0.2 | | 12.1 ± 0.0 | 11.4 ± 0.3 |
| **BW (g)** | 23.6 ± 0.5 | 22.1± 0.3 | 22.1 ± 0.9 | 27.5 ± 1.6 | | 25.7 ± 0.5 | 26.1 ± 0.9 |
| **Heart rate (bpm)** | 588 ± 35 | 556 ± 18 | 590 ± 15 | 577 ± 11 | | 592 ± 19 | 529 ± 14* |
| **LVIDd (mm)** | 3.32 ± 0.04 | 3.29 ± 0.5 | 3.22 ± 0.06 | 3.81 ±0.10 | | 3.77 ± 0.08 | 3.77 ± 0.14 |
| **LVIDs (mm)** | 2.00 ± 0.06 | 1.92 ± 0.05 | 1.93 ± 0.06 | 2.46 ± 0.07 | | 2.54 ± 0.09 | 2.73 ± 0.15 |
| **IVSd (mm)** | 0.74 ± 0.01 | 0.78 ± 0.01 | 0.78 ± 0.01 | 0.94 ± 0.04 | | 0.98 ± 0.03 | 1.00 ± 0.03 |
| **IVSs (mm)** | 1.19 ± 0.02 | 1.24 ± 0.02 | 1.15 ± 0.03 | 1.34 ± 0.03 | | 1.37 ± 0.01 | 1.40 ± 0.04 |
| **LVPWd (mm)** | 0.78 ± 0.02 | 0.82 ± 0.01 | 0.78 ± 0.02 | 0.90 ± 0.04 | | 0.96 ± 0.03 | 0.97 ± 0.04 |
| **LVPWs (mm)** | 1.22 ± 0.03 | 1.22 ± 0.02 | 1.15 ± 0.03 | 1.36 ± 0.06 | | 1.39 ± 0.02 | 1.36 ± 0.03 |
| **FS (%)** | 40.1 ± 1.14 | 41.6 ± 0.8 | 40.0 ± 1.3 | 35.5 ± 1.4 | | 32.7 ± 1.3 | 27.8 ± 2.1* |
| **EF (%)** | 71.8 ± 1.40 | 73.6 ± 0.9 | 71.7 ± 1.5 | 65.6 ± 1.8 | | 61.8 ± 1.9 | 54.2 ± 3.3* |
| **LVM/BW (mg/g)** | 3.50 ± 0.07 | 3.92 ± 0.07*** | 3.69 ± 0.12 | 4.86 ± 0.33 | | 5.59 ± 0.27 | 5.59 ± 0.38 |

All data are presented as mean ± standard error of the mean (SEM). M, months; BW, body weight; LVID, left ventricular inner diameter; IVS, interventricular septum; LVPW, left ventricular posterior wall thickness; FS, fractional shortening; EF, ejection fraction; LVM, left ventricular mass; bpm, beats per minute; d, diastole; s, systole. **P* < 0.05, ****P* < 0.001 *vs*. *Palld*^fl/fl^; linear mixed model with Tukey’s multiple comparisons test.
