# Supplementary material for: Ablation of palladin in adult heart causes dilated cardiomyopathy associated with intercalated disc abnormalities
Source: eLife. 2023 Mar 16;12:e78629. doi: 10.7554/eLife.78629 (PMC10069870; doi:10.7554/eLife.78629)
Supplement: Figure 2—source data 6. [file elife-78629-fig2-data6.docx]

**Figure 2–source data 6.** Echocardiographic parameters of 3- and 6-month-old cardiomyocyte-specific palladin and myopalladin double knockout (cPKO/MKO dKO) mice subjected to transaortic constriction (TAC) or SHAM.

|  | **2M** | | | | |
| --- | --- | --- | --- | --- | --- |
|  | **WT**  **(*n* = 18)** | ***Palld^fl/fl^;Myh6^Cre/+^***  **(*n* = 16)** | ***Mypn^-/-^***  **(*n* = 23)** | ***Mypn^-/-^*/*Palld^fl/fl^***  **(*n* = 6)** | ***Mypn*^-/-^/**  ***Palld*^fl/fl^;*Myh6^Cre/^*^+^**  **(*n* = 7)** |
| **Body weight (g)** | 24.9 ± 0.5 | 27.1 ± 0.8 | 23.9 ± 0.3*^,δδδ^ | 22.7 ± 0.7^δδδ^ | 23.1 ± 0.5^δδδ^ |
| **Heart rate (bpm)** | 603 ± 14 | 591 ± 21 | 574 ± 18 | 587 ± 11 | 566 ± 27 |
| **LVIDd (mm)** | 3.40 ± 0.05 | 3.46 ± 0.06 | 3.56 ± 0.03 | 3.36 ± 0.10 | 3.54 ± 0.08 |
| **LVIDs (mm)** | 2.08 ± 0.04 | 2.17 ± 0.05 | 2.26 ± 0.03* | 2.11 ± 0.10 | 2.33 ± 0.07* |
| **IVSd (mm)** | 0.78 ± 0.01 | 0.79 ± 0.02 | 0.79 ± 0.01 | 0.86 ± 0.01* | 0.84 ± 0.01 |
| **IVSs (mm)** | 1.17 ± 0.01 | 1.18 ± 0.02 | 1.20 ± 0.02 | 1.22 ± 0.01 | 1.18 ± 0.02 |
| **LVPWd (mm)** | 0.77 ± 0.02 | 0.81 ± 0.02 | 0.78 ± 0.02 | 0.76 ± 0.02 | 0.77 ± 0.02 |
| **LVPWs (mm)** | 1.21 ± 0.01 | 1.21 ± 0.02 | 1.18 ± 0.01 | 1.14 ± 0.03 | 1.12 ± 0.03*^,δ^ |
| **FS (%)** | 38.9 ± 0.4 | 37.3 ± 0.5 | 36.7 ± 0.4 | 37.5 ± 1.4 | 34.3 ± 1.3*** |
| **EF (%)** | 70.5 ± 0.6 | 68.3 ± 0.7 | 67.5 ± 0.6 | 68.6 ± 1.9 | 64.2 ± 1.8*** |
| **LVM/BW (mg/g)** |  |  |  | 4.07 ± 0.22 | 4.31 ± 0.18 |
|  | **4M** | | | | |
|  | **WT**  **(*n* = 42)** | ***Palld^fl/fl^;Myh6^Cre/+^***  **(*n* = 7)** | ***Mypn^-/-^***  **(*n* = 27)** | ***Mypn^-/-^*/*Palld^fl/fl^***  **(*n* = 6)** | ***Mypn*^-/-^/**  ***Palld*^fl/fl^;*Myh6^Cre/^*^+^**  **(*n* = 7)** |
| **Body weight (g)** | 29.2 ± 0.3 | 24.1 ± 1.0*** | 26.4 ± 0.3***^,δ^ | 30.7 ± 1.4^δδδ^ | 30.9 ± 1.0^δδδ^ |
| **Heart rate (bpm)** | 597 ± 8 | 661± 33 | 560 ± 10^δδ^ | 554 ± 28^δ^ | 541 ± 29^δδ^ |
| **LVIDd (mm)** | 3.41 ± 0.03 | 3.37 ± 0.05 | 3.87 ± 0.04***^,δδδ^ | 3.75 ± 0.10**^,δδ^ | 3.71 ± 0.09**^,δ^ |
| **LVIDs (mm)** | 2.05 ± 0.03 | 2.09 ± 0.04 | 2.70 ± 0.04***^,δδδ^ | 2.53 ± 0.10***^,δδδ^ | 2.58 ± 0.12***^,δδδ^ |
| **IVSd (mm)** | 0.82 ± 0.01 | 0.81 ± 0.02 | 0.83 ± 0.01 | 0.85 ± 0.01 | 0.83 ± 0.03 |
| **IVSs (mm)** | 1.25 ± 0.02 | 1.22 ± 0.02 | 1.19 ± 0.02* | 1.24 ± 0.02 | 1.18 ± 0.02 |
| **LVPWd (mm)** | 0.82 ± 0.01 | 0.77 ± 0.02 | 0.83 ± 0.01 | 0.80 ± 0.02 | 0.82 ± 0.04 |
| **LVPWs (mm)** | 1.22 ± 0.01 | 1.16 ± 0.03 | 1.19 ± 0.02** | 1.22 ± 0.03 | 1.14 ± 0.03* |
| **FS (%)** | 40.0 ± 0.4 | 38.0 ± 0.4 | 30.2 ± 0.4***^,δδδ^ | 32.7 ± 1.4***^,δδ^ | 30.5 ± 1.9***^,δδδ^ |
| **EF (%)** | 71.7 ± 0.5 | 69.4 ± 0.5 | 58.2 ± 0.6***^,δδδ^ | 61.8 ± 2.1***^,δδ^ | 58.5 ± 2.8***^,δδδ^ |
| **LVM/BW (mg/g)** |  |  |  | 3.67 ± 0.13 | 3.62 ± 0.20 |

All data are presented as mean ± standard error of the mean (SEM). M, months; LVID, left ventricular inner diameter; IVS, interventricular septum; LVPW, left ventricular posterior wall thickness; FS, fractional shortening; EF, ejection fraction; BW, body weight; HW, heart weight; bpm, beats per minute; d, diastole; s, systole. **P* < 0.05, ***P* < 0.01, ****P* < 0.001 *vs*. WT; ^δ^*P* < 0.05, ^δδ^*P* < 0.01, ^δδδ^*P* < 0.001 *vs*. *Palld*^fl/fl^;Cre^+/0^; linear mixed model with Tukey’s multiple comparisons test.
