# Supplementary material for: Ablation of palladin in adult heart causes dilated cardiomyopathy associated with intercalated disc abnormalities
Source: eLife. 2023 Mar 16;12:e78629. doi: 10.7554/eLife.78629 (PMC10069870; doi:10.7554/eLife.78629)
Supplement: Figure 2—figure supplement 2—source data 1. [file elife-78629-fig2-figsupp2-data1.docx]

|  | **3M** | | | **6M** | | |
| --- | --- | --- | --- | --- | --- | --- |
|  | ***Palld^fl/fl^***  **(*n* = 17)** | ***Myh6^Cre/+^***  **(*n* = 7)** | ***Palld^fl/fl^; Myh6^Cre/+^***  **(*n* = 16)** | ***Palld^fl/fl^***  **(*n* = 11)** | ***Myh6^Cre/+^***  **(*n* = 8)** | ***Palld^fl/fl^; Myh6^Cre/+^***  **(*n* = 15)** |
| **BW (g)** | 27.1 ± 0.5 | 27.3 ± 0.9 | 27.1 ± 0.8 | 34.5 ± 4.6 | 32.9 ± 1.3 | 33.3 ± 0.8 |
| **Heart rate (bpm)** | 559 ± 16 | 572 ± 21 | 591 ± 21 | 661 ± 52 | 577 ± 18 | 608 ± 11 |
| **LVIDd (mm)** | 3.40 ± 0.06 | 3.63 ± 0.06* | 3.46 ± 0.06 | 3.50 ± 0.04 | 3.59 ± 0.05 | 3.44 ± 0.05 |
| **LVIDs (mm)** | 2.09 ± 0.04 | 2.32 ± 0.07* | 2.17 ± 0.05 | 2.17 ± 0.05 | 2.21 ± 0.06 | 2.14 ± 0.04 |
| **IVSd (mm)** | 0.84 ± 0.02 | 0.86 ± 0.04 | 0.79 ± 0.02 | 0.82 ± 0.02 | 0.88 ± 0.03 | 0.82 ± 0.02 |
| **IVSs (mm)** | 1.30 ± 0.03 | 1.31 ± 0.04 | 1.18 ± 0.02**^,δ^ | 1.25 ± 0.02 | 1.29 ± 0.02 | 1.18 ± 0.02 |
| **LVPWd (mm)** | 0.83 ± 0.02 | 0.84 ± 0.02 | 0.81 ± 0.02 | 0.83 ± 0.03 | 0.82 ± 0.01 | 0.81 ± 0.02 |
| **LVPWs (mm)** | 1.26 ± 0.02 | 1.25 ± 0.03 | 1.21 ± 0.02 | 1.22 ± 0.02 | 1.26 ± 0.02 | 1.24 ± 0.02 |
| **FS /%)** | 38.4 ± 0.7 | 36.3 ± 1.1 | 37.3 ± 0.5 | 38.2 ± 0.7 | 38.6 ± 1.1 | 37.7 ± 0.7 |
| **EF (%)** | 69.7 ± 0.8 | 66.8 ± 1.4 | 68.3 ± 0.7 | 69.4 ± 0.9 | 69.8 ± 1.3 | 68.9 ± 0.9 |
| **LVM (mg)** | 97.2 ± 3.7 | 112.1 ± 6.8 | 94.7 ± 4.0 | 100.9 ± 3.5 | 109.7 ± 3.3 | 96.5 ± 3.8 |
| **LVM/BW (mg/g)** | 3.59 ± 0.12 | 4.11 ± 0.23 | 3.51 ± 0.12 | 2.97 ± 0.15 | 3.37 ± 0.15 | 2.91 ± 0.12 |

**Figure 2–figure supplement 2–source data 1.** Echocardiographic parameters of 3- and 6-month-old cardiomyocyte-specific palladin knockout (cPKO) male mice compared to controls under basal conditions.

All data are presented as mean ± standard error of the mean (SEM). M, months; BW, body weight; LVID, left ventricular inner diameter; IVS, interventricular septum; LVPW, left ventricular posterior wall thickness; FS, fractional shortening; EF, ejection fraction; LVM, left ventricular mass; bpm, beats per minute; d, diastole; s, systole. **P* < 0.05, ***P* < 0.01 *vs*. *Palld*^fl/fl^; ^δ^*P* < 0.05 *vs*. Cre^+/0^; linear mixed model with Tukey’s multiple comparisons test.
