# Supplementary material for: Ablation of palladin in adult heart causes dilated cardiomyopathy associated with intercalated disc abnormalities
Source: eLife. 2023 Mar 16;12:e78629. doi: 10.7554/eLife.78629 (PMC10069870; doi:10.7554/eLife.78629)
Supplement: Figure 3—source data 3. [file elife-78629-fig3-data3.docx]

**Figure 3–source data 3.** Echocardiographic parameters of inducible cardiomyocyte-specific palladin knockout (cPKOi) male mice compared to controls 8 and 24 weeks after tamoxifen (TAM) induction.

|  | **8W** | | | |
| --- | --- | --- | --- | --- |
|  | ***Palld^fl/fl^* TAM**  **(*n* = 30)** | ***Myh6^MCM/+^* TAM**  **(*n* = 25)** | ***Palld^fl/fl^;Myh6^MCM/+^***  **(*n* = 7)** | ***Palld^fl/fl^;Myh6^MCM/+^* TAM (*n* = 44)** |
| **Body weight (g)** | 29.4 ± 0.6 | 28.4 ± 2.5 | 28.7 ± 0.6 | 29.6 ± 0.5 |
| **Heart rate (bpm)** | 553 ± 11 | 588 ± 10 | 526 ± 16^δ^ | 547 ± 9^δ^ |
| **LVIDd (mm)** | 3.54 ± 0.03 | 3.57 ± 0.03 | 3.60 ± 0.05 | 3.90 ± 0.04***^,δδδ,ηη^ |
| **LVIDs (mm)** | 2.18 ± 0.03 | 2.24 ± 0.03 | 2.29 ± 0.06 | 2.77 ± 0.04***^,δδδ,ηηη^ |
| **IVSd (mm)** | 0.84 ± 0.01 | 0.82 ± 0.01 | 0.83 ± 0.03 | 0.86 ± 0.01* |
| **IVSs (mm)** | 1.24 ± 0.01 | 1.20 ± 0.01 | 1.29 ± 0.02^δδ^ | 1.22 ± 0.01^η^ |
| **LVPWd (mm)** | 0.80 ± 0.01 | 0.82 ± 0.02 | 0.85 ± 0.05 | 0.82 ± 0.02 |
| **LVPWs (mm)** | 1.23 ± 0.01 | 1.22 ± 0.01 | 1.22 ± 0.03 | 1.16 ± 0.02**^,δ^ |
| **FS (%)** | 38.5 ± 0.5 | 37.5 ± 0.5 | 36.5 ± 0.9 | 29.0 ± 0.5***^,δδδ, ηηη^ |
| **EF (%)** | 69.7 ± 0.6 | 68.4 ± 0.6 | 67.2 ± 1.2 | 56.3 ± 0.7***^,δδδ, ηηη^ |
| **HW/BW (mg/g)** | 4.91 ± 0.41 (*n* = 19) | 5.15 ± 0.44 (*n* = 15) |  | 5.08 ± 0.59 (*n* = 27) |
|  | **24W** | | | |
|  | ***Palld^fl/fl^* TAM**  **(*n* = 9)** | ***Myh6^MCM/+^* TAM**  **(*n* = 5)** | ***Palld^fl/fl^;Myh6^MCM/+^***  **(*n* = 4)** | ***Palld^fl/fl^;Myh6^MCM/+^* TAM (*n* = 8)** |
| **Body weight (g)** | 32.1 ± 1.2 | 31.0 ± 4.6 | 36.0 ± 1.5 | 33.0 ± 1.8 |
| **Heart rate (bpm)** | 593 ± 19 | 575 ± 22 | 557 ± 4.2***^,δδδ^ | 570 ± 27^ηηη^ |
| **LVIDd (mm)** | 3.57 ± 0.05 | 3.62 ± 0.03 | 3.64 ± 0.06 | 4.00 ± 0.06***^,δδδ,η^ |
| **LVIDs (mm)** | 2.20 ± 0.05 | 2.28 ± 0.03 | 2.25 ± 0.09 | 2.94 ± 0.08***^,δδδ,ηη^ |
| **IVSd (mm)** | 0.82 ± 0.02 | 0.81 ± 0.02 | 0.87 ± 0.02 | 0.92 ± 0.02**^,δ^ |
| **IVSs (mm)** | 1.25 ± 0.03 | 1.25 ± 0.03 | 1.30 ± 0.01 | 1.20 ± 0.02^η^ |
| **LVPWd (mm)** | 0.82 ± 0.02 | 0.83 ± 0.04 | 0.85 ± 0.00 | 0.81 ± 0.02 |
| **LVPWs (mm)** | 1.23 ± 0.03 | 1.22 ± 0.04 | 1.25 ± 0.01 | 1.15 ± 0.04 |
| **FS (%)** | 38.4 ± 0.5 | 36.8 ± 0.8 | 38.4 ± 0.5***^,δδδ,ηηη^ | 26.6 ± 1.2***^,δδδ,ηη^ |
| **EF (%)** | 69.7 ± 0.7 | 67.6 ± 1.1 | 69.5 ± 1.9***^,δδδ,ηηη^ | 52.5 ± 2.0***^,δδδ,ηη^ |
| **HW/BW (mg/g)** | 4.68 ± 0.60 (*n* = 11) | 5.23 ± 0.37 (*n* = 7) |  | 4.89 ± 0.58 (*n* = 13) |

All data are presented as mean ± standard error of the mean (SEM). M, months; LVID, left ventricular inner diameter; IVS, interventricular septum; LVPW, left ventricular posterior wall thickness; FS, fractional shortening; EF, ejection fraction; BW, body weight; HW, heart weight; bpm, beats per minute; d, diastole; s, systole. **P* < 0.05, ***P* < 0.01, ****P* < 0.001 *vs*. *Palld*^fl/fl^ TAM; ^δ^*P* < 0.05, ^δδδ^*P* < 0.001 *vs*. MCM^+/0^ TAM; ^η^*P* < 0.05, ^ηη^*P* < 0.01, ^ηηη^*P* < 0.001 *vs*. *Palld*^fl/fl^;MCM^+/0^ TAM; linear mixed model with Tukey’s multiple comparisons test.
