# Supplementary material for: Ablation of palladin in adult heart causes dilated cardiomyopathy associated with intercalated disc abnormalities
Source: eLife. 2023 Mar 16;12:e78629. doi: 10.7554/eLife.78629 (PMC10069870; doi:10.7554/eLife.78629)
Supplement: Figure 3—figure supplement 1—source data 1. [file elife-78629-fig3-figsupp1-data1.docx]

**Figure 3-figure supplement 1-source data 1.** Echocardiographic parameters of cardiomyocyte-specific palladin knockout (cPKO) male mice compared to controls 8 weeks after tamoxifen (TAM) injection.

|  | ***Myh6^Cre/+^* TAM (*n* = 7)** | ***Palld^fl/fl^;Myh6^Cre/+^* TAM (*n* = 7)** |
| --- | --- | --- |
| **Body weight (g)** | 26.1 ± 1.4 | 19.1 ± 0.4 |
| **Heart rate (bpm)** | 622 ± 25 | 693 ± 43 |
| **LVIDd (mm)** | 3.43 ± 0.07 | 3.37 ± 0.05 |
| **LVIDs (mm)** | 2.10 ± 0.05 | 2.09 ± 0.04 |
| **IVSd (mm)** | 0.81 ± 0.03 | 0.81 ± 0.02 |
| **IVSs (mm)** | 1.22 ± 0.03 | 1.22 ± 0.02 |
| **LVPWd (mm)** | 0.82 ± 0.03 | 0.77 ± 0.02 |
| **LVPWs (mm)** | 1.19 ± 0.03 | 1.16 ± 0.03 |
| **FS (%)** | 38.9 ± 1.2 | 38.0 ± 0.4 |
| **EF (%)** | 70.3 ± 1.4 | 69.4 ± 0.5 |
| **LVM (mg)** | 96.1 ± 5.7 | 88.7 ± 3.7 |
| **LVM/BW (mg/g)** | 36.8 ± 1.4 | 36.9 ± 1.5 |

All data are presented as mean ± standard error of the mean (SEM). LVID, left ventricular inner diameter; IVS, interventricular septum; LVPW, left ventricular posterior wall thickness; FS, fractional shortening; EF, ejection fraction; LVM, left ventricular mass; BW, body weight; bpm, beats per minute; d, diastole; s, systole. No differences using linear mixed model with Tukey’s multiple comparisons test.
