# Supplementary material for: Ablation of palladin in adult heart causes dilated cardiomyopathy associated with intercalated disc abnormalities
Source: eLife. 2023 Mar 16;12:e78629. doi: 10.7554/eLife.78629 (PMC10069870; doi:10.7554/eLife.78629)
Supplement: Figure 4—source data 4. [file elife-78629-fig4-data4.docx]

**Figure 4–Source data 4.** Quantitative real-time PCR (qRT-PCR) analysis on left ventricle from inducible cardiomyocyte-specific palladin knockout (cPKOi) male mice compared to controls 6 months after tamoxifen (TAM) induction.

| **Gene** | ***Pall^fl/fl^* TAM**  **(*n* = 5)** | ***Myh6^MCM/+^* TAM**  **(*n* = 5)** | ***Pall^fl/fl^;Myh6^MCM/+^* TAM**  **(*n* = 4)** |
| --- | --- | --- | --- |
| ***Nppa*** | 0.85 ± 0.28 | 1.00 ± 0.10 | 1.08 ± 0.25 |
| ***Nppb*** | 0.80 ± 0.12 | 1.00 ± 0.07 | 1.58 ± 0.21**^,δ^ |
| ***Myh6*** | 0.67 ± 0.06 | 1.00 ± 0.05 | 0.72 ± 0.06 |
| ***Myh7*** | 5.52 ± 1.96 | 1.00 ± 0.14** | 14.61 ± 2.47***^,δδδ^ |
| ***Actc1*** | 0.97 ± 0.07 | 1.00 ± 0.05 | 1.01 ± 0.09 |
| ***Acta1*** | 0.50 ± 0.12 | 1.00 ± 0.10* | 0.73 ± 0.10 |
| ***Ankrd1*** | 0.98 ± 0.09 | 1.00 ± 0.06 | 1.62 ± 0.23*^,δ^ |
| ***Nebl*** | 0.87 ± 0.06 | 1.00 ± 0.09 | 0.89 ± 0.08 |
| ***Ldb3*** | 1.19± 0.33 | 1.00 ± 0.23 | 2.03 ± 0.34 |
| ***Pdlim3*** | 1.19 ± 0.08 | 1.00 ± 0.20 | 1.45 ± 0.15 |
| ***Des*** | 0.95 ± 0.03 | 1.00 ± 0.03 | 0.92 ± 0.04 |
| ***Tnnc1*** | 1.06 ± 0.02 | 1.00 ± 0.03 | 1.07 ± 0.08 |
| ***Atp2a2*** | 0.96 ± 0.05 | 1.00 ± 0.06 | 0.89 ± 0.07 |
| ***Srf*** | 1.10 ± 0.09 | 1.00 ± 0.05 | 1.16 ± 0.09 |
| ***Mk11*** | 0.86 ± 0.03 | 1.00 ± 0.04 | 0.96 ± 0.07 |
| ***Col1a1*** | 0.81 ± 0.06 | 1.00 ± 0.05 | 1.11 ± 0.16 |
| ***Col3a1*** | 0.95 ± 0.10 | 1.00 ± 0.08 | 0.96 ± 0.15 |
| ***Ctgf/Ccn2*** | 1.02 ± 0.12 | 1.00 ± 0.11 | 1.37 ± 0.18 |
| ***Acta2*** | 0.79 ± 0.11 | 1.00 ± 0.12 | 0.59 ± 0.11 |
| ***Tgfb1*** | 0.92 ± 0.06 | 1.00 ± 0.10 | 0.97 ± 0.06 |
| ***Bcl2*** | 0.86 ± 0.08 | 1.00 ± 0.10 | 1.40 ± 0.10**^,δ^ |
| ***Bax*** | 0.90 ± 0.07 | 1.00 ± 0.06 | 1.12± 0.10 |
| ***Tp53*** | 0.88 ± 0.05 | 1.00 ± 0.09 | 1.06 ± 0.09 |
| ***Egr1*** | 0.76 ± 0.24 | 1.00 ± 0.11 | 1.56 ± 0.36 |

All values are presented as mean ± standard error of the mean (SEM). **P* < 0.05, ***P* < 0.01 *vs*. *Palld*^fl/fl^; ^δ^*P* < 0.05, ^δδδ^*P* < 0.001 *vs*. Cre^+/0^; one-way analysis of variance (ANOVA) with Tukey’s multiple comparisons test.
