# Supplementary material for: Ablation of palladin in adult heart causes dilated cardiomyopathy associated with intercalated disc abnormalities
Source: eLife. 2023 Mar 16;12:e78629. doi: 10.7554/eLife.78629 (PMC10069870; doi:10.7554/eLife.78629)
Supplement: Figure 6—source data 6. [file elife-78629-fig6-data6.docx]

**Figure 6–source data 6.** Patient characteristics.

| **Number** | **Gender** | **Age (years)** | **Disease** | **qRT-PCR** | **WB** |
| --- | --- | --- | --- | --- | --- |
| 1 | M | 20-50 | NF | x |  |
| 2 | M | 20-50 | NF | x |  |
| 3 | M | 20-50 | NF | x |  |
| 4 | M | 20-50 | NF | x |  |
| 5 | M | 20-50 | NF | x |  |
| 6 | M | 66 | NF | x | x |
| 7 | M | 54 | NF | x | x |
| 8 | M | 41 | NF | x | x |
| 9 | M | 67 | NF | x | x |
| 10 | M | 57 | NF | x |  |
| 11 | M | 59 | NF | x |  |
| 12 | M | 62 | NF | x | x |
| 13 | M | 59 | NF | x | x |
| 14 | F | 59 | NF | x |  |
| 15 | F | 51 | NF | x |  |
| 16 | F | 30 | NF | x | x |
| 17 | M | 54 | DCM | x | x |
| 18 | M | 48 | DCM | x | x |
| 19 | M | 63 | DCM | x | x |
| 20 | M | 67 | DCM | x |  |
| 21 | M | 60 | DCM | x |  |
| 22 | M | 61 | DCM | x |  |
| 23 | M | 71 | DCM | x |  |
| 24 | M | 65 | DCM | x |  |
| 25 | M | 74 | DCM | x | x |
| 26 | M | 50-75 | DCM | x | x |
| 27 | M | 50-75 | DCM | x | x |
| 28 | M | 50-75 | DCM | x | x |
| 29 | M | 50-75 | DCM | x | x |
| 30 | M | 50-75 | DCM | x | x |
| 31 | M | 50-75 | DCM | x | x |
| 32 | M | 58 | ICM | x | x |
| 33 | M | 50 | ICM | x |  |
| 34 | M | 56 | ICM | x |  |
| 35 | M | 60 | ICM | x |  |
| 36 | M | 53 | ICM | x |  |
| 37 | M | 67 | ICM | x |  |
| 38 | M | 58 | ICM | x |  |
| 39 | M | 58 | ICM | x |  |
| 40 | M | 56 | ICM | x | x |
| 41 | M | 60 | ICM | x | x |
| 42 | M | 50-75 | ICM | x | x |
| 43 | M | 50-75 | ICM | x | x |
| 44 | M | 50-75 | ICM | x | x |
| 45 | M | 50-75 | ICM | x | x |
| 46 | M | 50-75 | ICM | x | x |

M, male; NF, non failing; DCM, dilated cardiomyopathy; ICM, ischemic cardiomyopathy; qRT-PCR, quantitative real-time blot; WB, Western blot analysis.
