# Supplementary material for: Ablation of palladin in adult heart causes dilated cardiomyopathy associated with intercalated disc abnormalities
Source: eLife. 2023 Mar 16;12:e78629. doi: 10.7554/eLife.78629 (PMC10069870; doi:10.7554/eLife.78629)
Supplement: Figure 6—figure supplement 3—source data 1. [file elife-78629-fig6-figsupp3-data1.pdf]

Figure 6–figure supplement 3-source data 1. Uncropped Western blots.

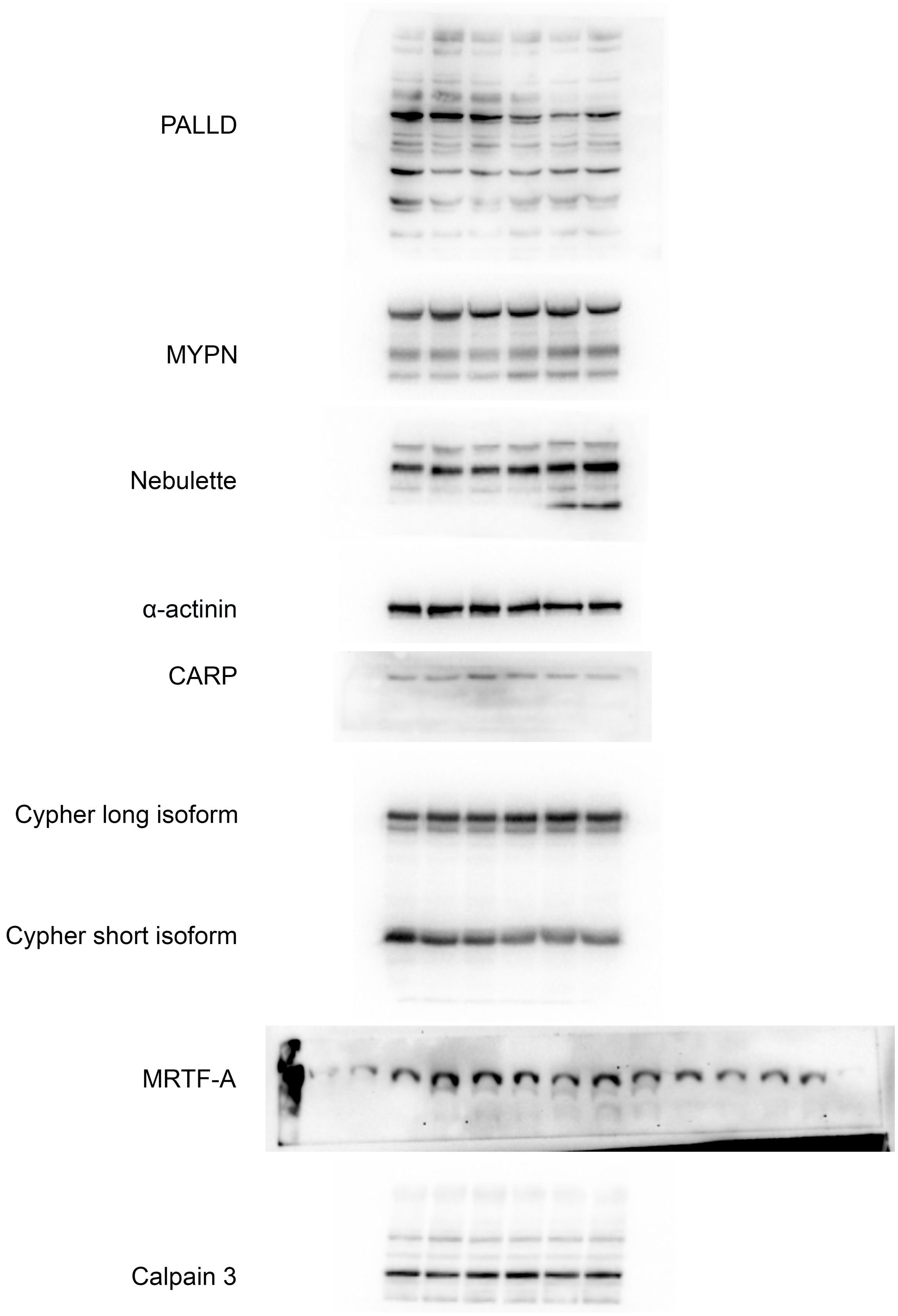

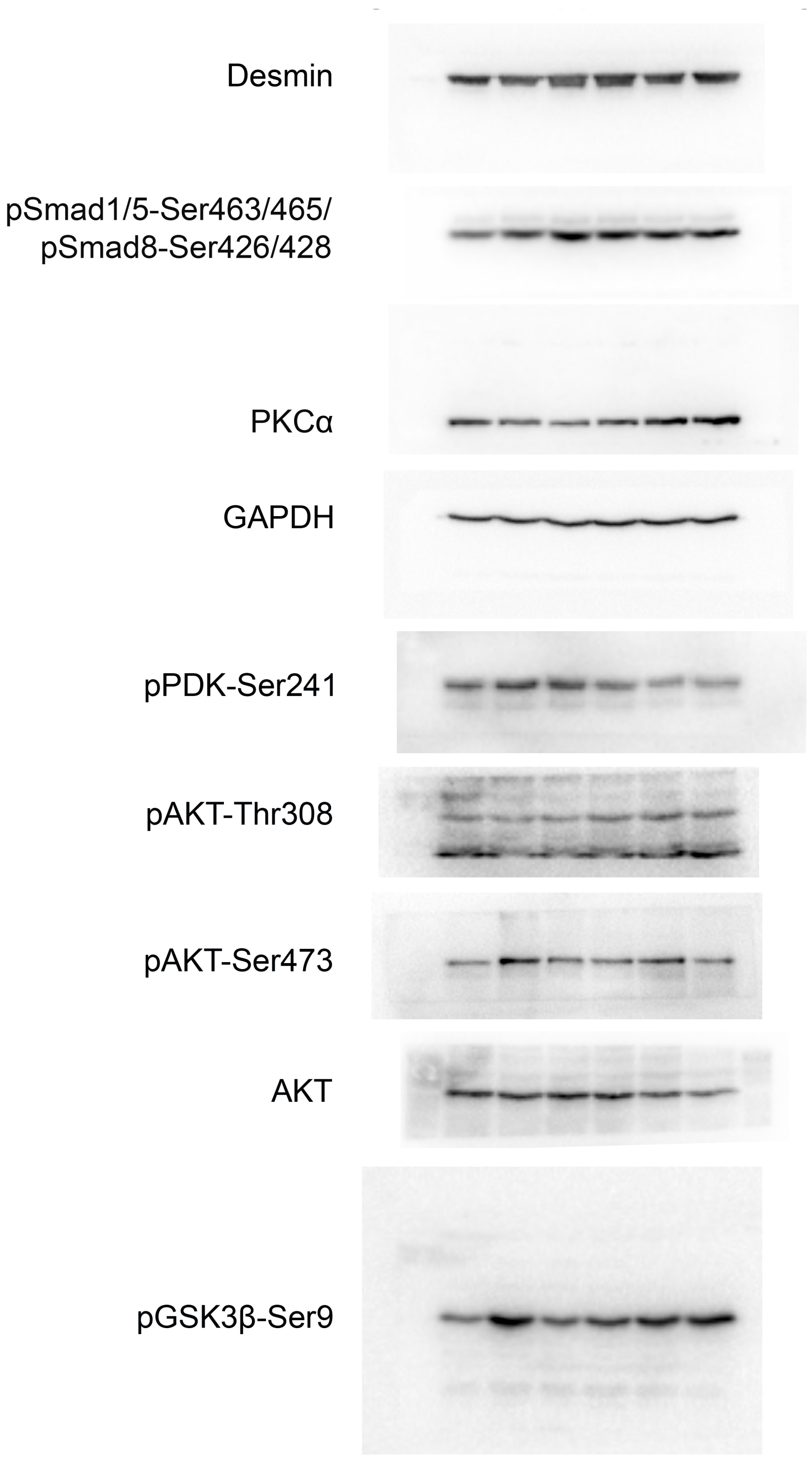
Desmin
pSmad1/5-Ser463/465/
pSmad8-Ser426/428
PKCα
GAPDH
pPDK-Ser241
pAKT-Thr308
pAKT-Ser473
AKT
pGSK3β-Ser9

GSK3β

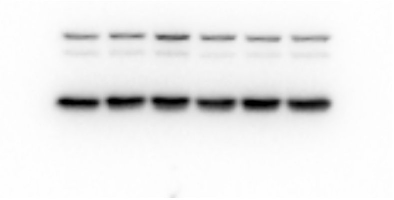

pP70S6K-Thr421/Ser424

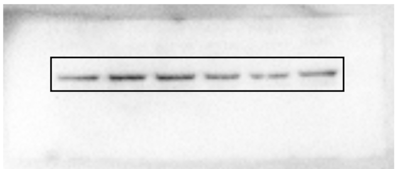

P70S6K

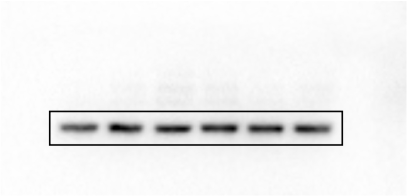

pMEK1/2-Ser217/221

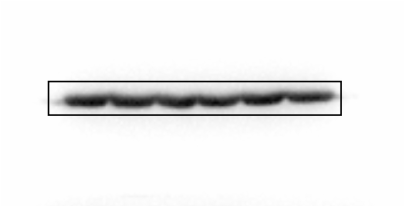

pErk1/2-Thr202/Tyr204

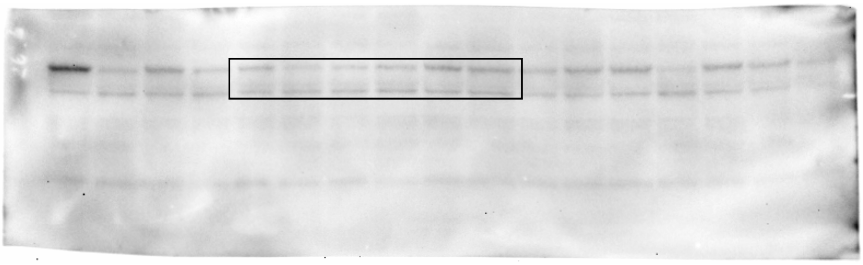

Erk1/2

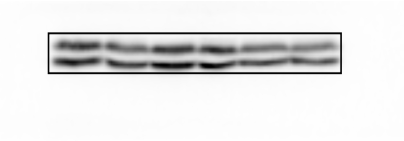

pP38-Thr180/Tyr182

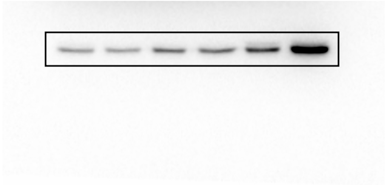

P38

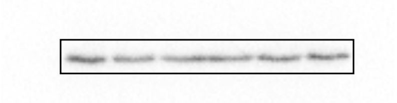

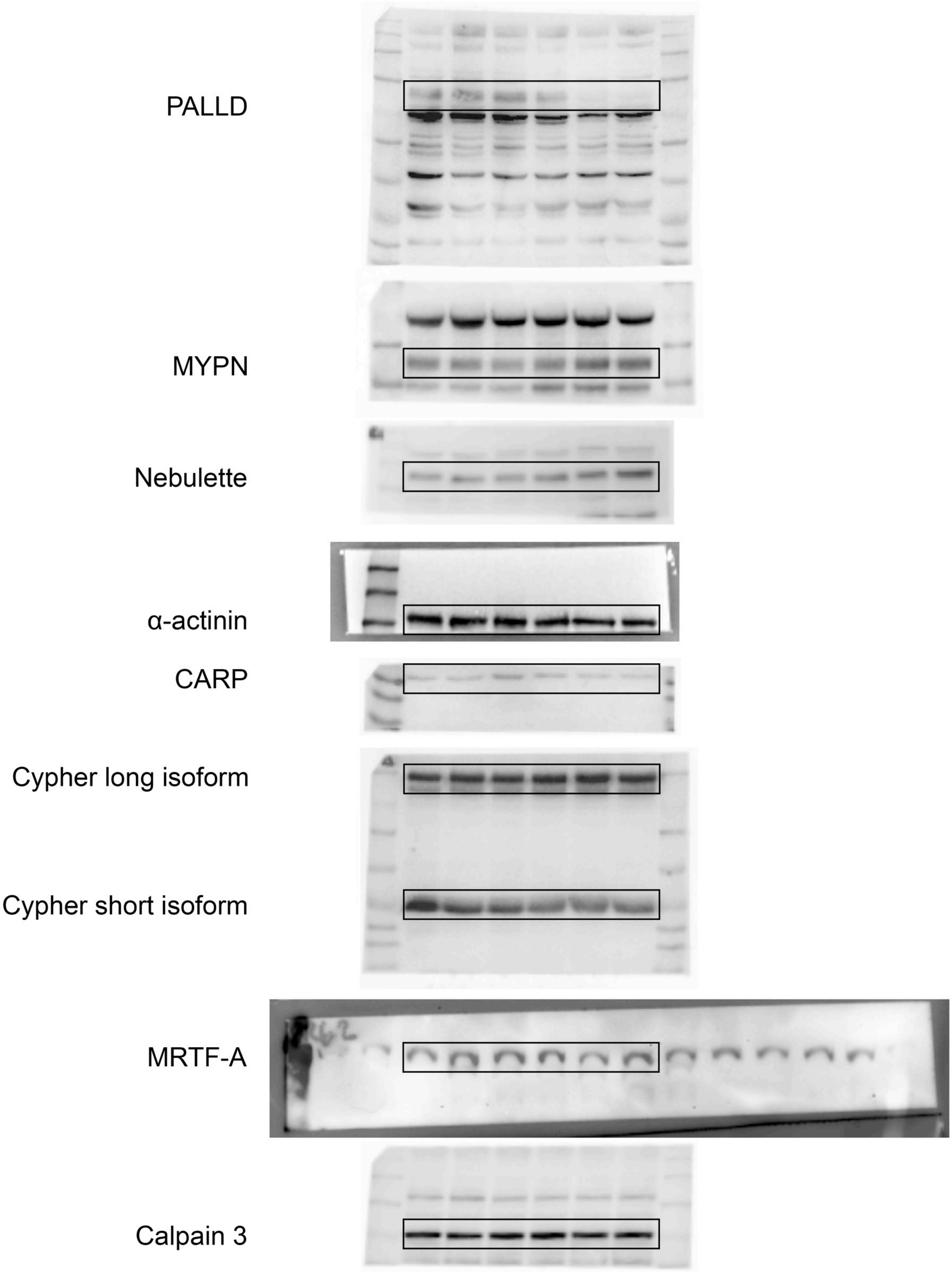
PALLD
MYPN
Nebulette
α-actinin
CARP
Cypher long isoform
Cypher short isoform
MRTF-A
Calpain 3

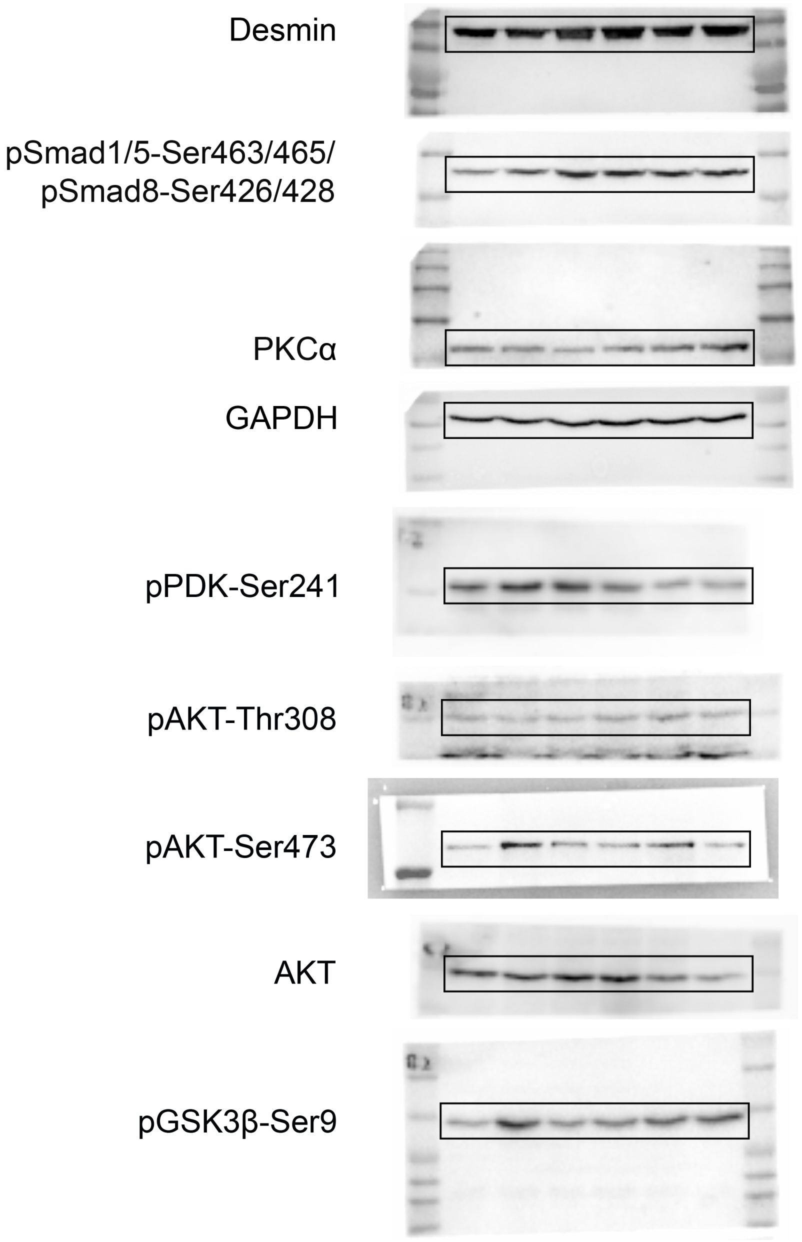
Desmin
pSmad1/5-Ser463/465/
pSmad8-Ser426/428
PKCα
GAPDH
pPDK-Ser241
pAKT-Thr308
pAKT-Ser473
AKT
pGSK3β-Ser9

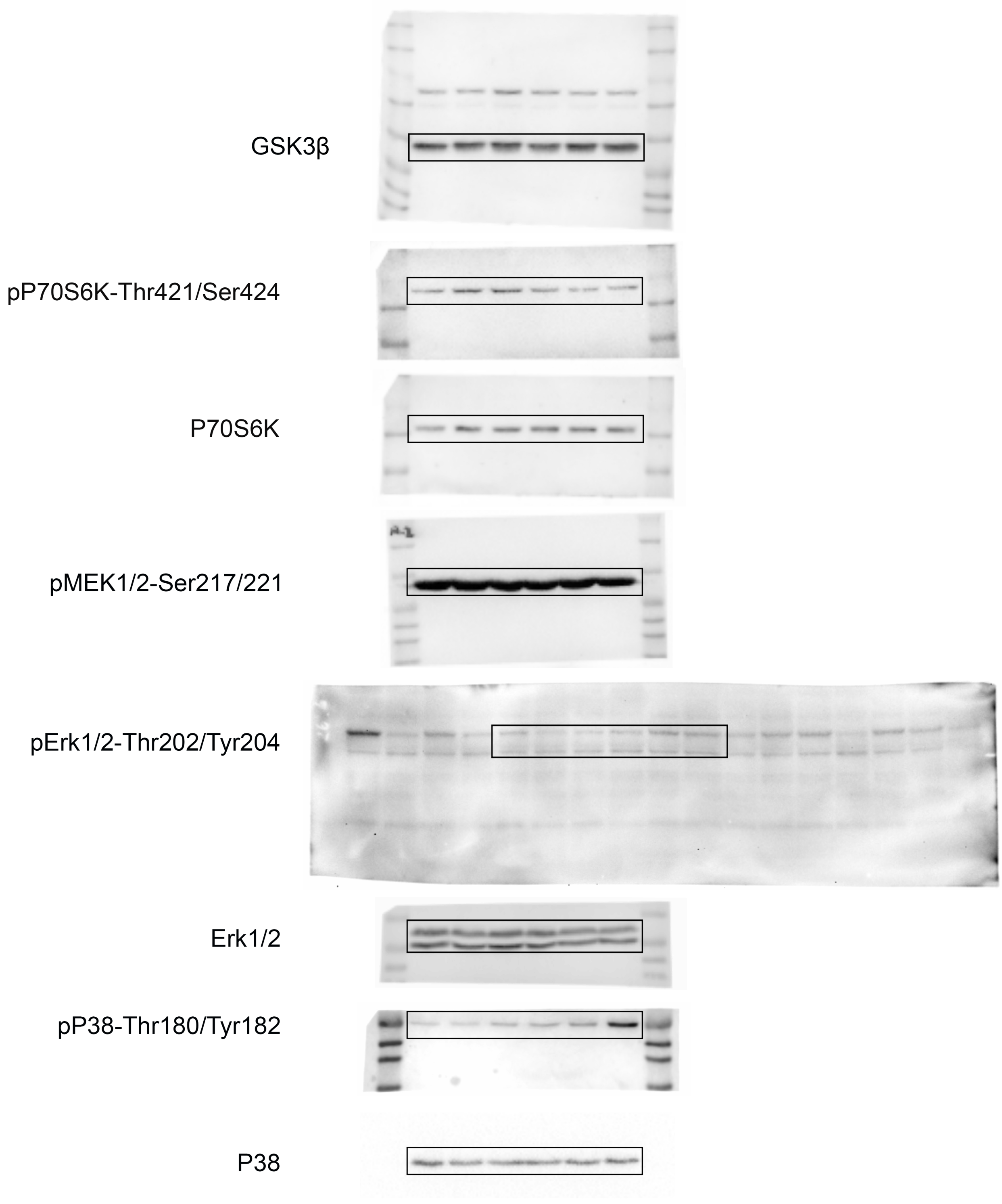
GSK3β
pP70S6K-Thr421/Ser424
P70S6K
pMEK1/2-Ser217/221
pErk1/2-Thr202/Tyr204
Erk1/2
pP38-Thr180/Tyr182
P38
