# Supplementary material for: Ablation of palladin in adult heart causes dilated cardiomyopathy associated with intercalated disc abnormalities
Source: eLife. 2023 Mar 16;12:e78629. doi: 10.7554/eLife.78629 (PMC10069870; doi:10.7554/eLife.78629)

Figure 6–figure supplement 4–source data 1. Uncropped Western blots.

Coomassie

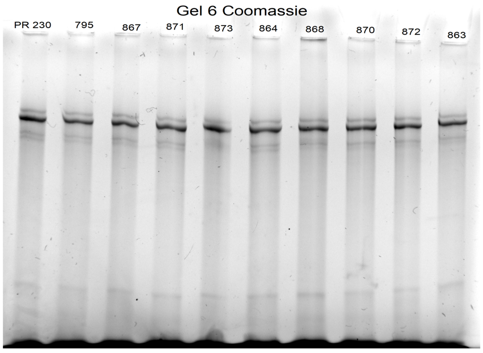

pTTN-Ser3991

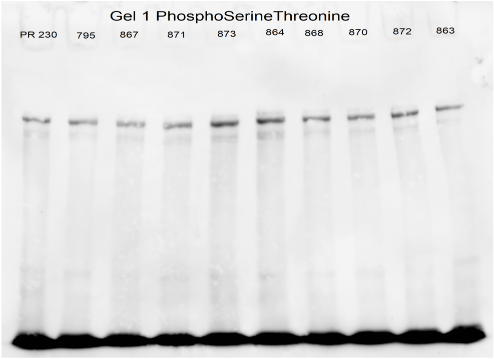

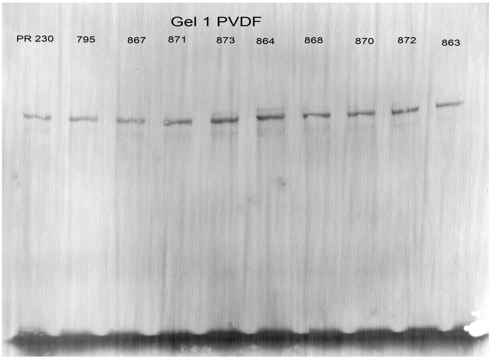

pTTN-Ser/Thr

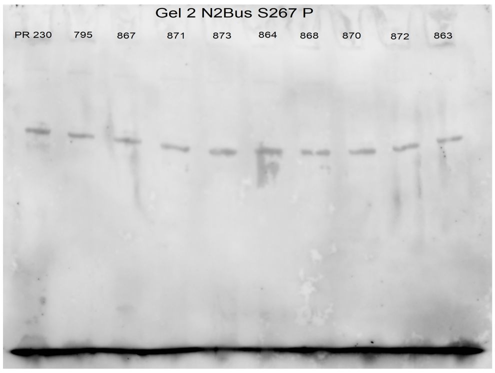

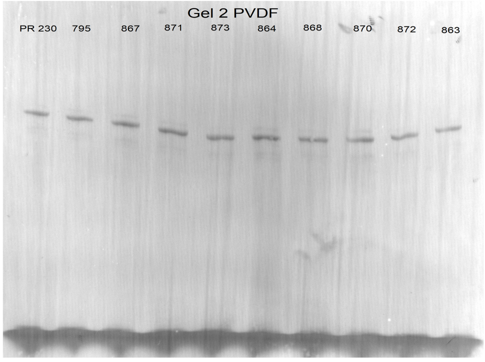

pTTN-Ser4080

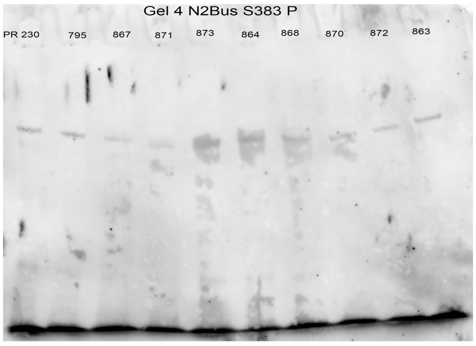

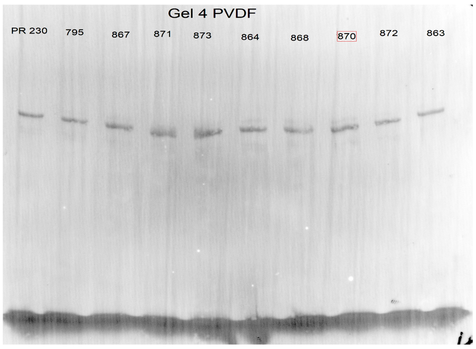

pTTN-Ser12742

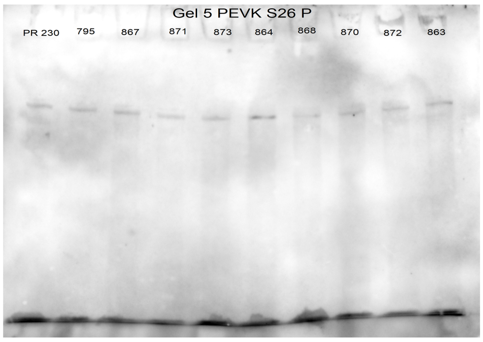

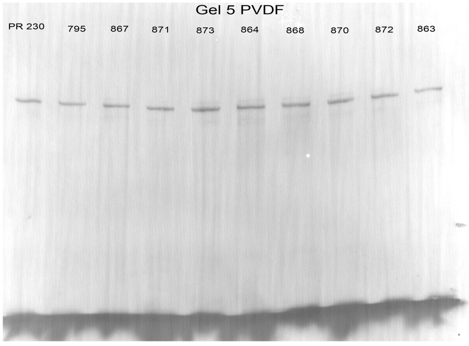

Coomassie

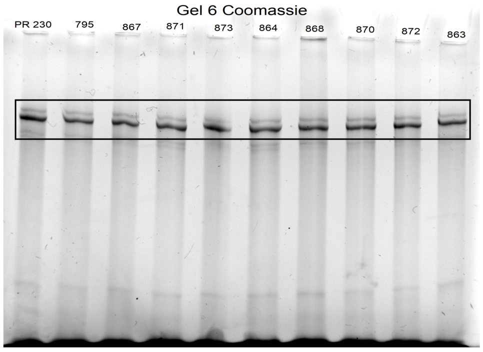

pTTN-Ser3991

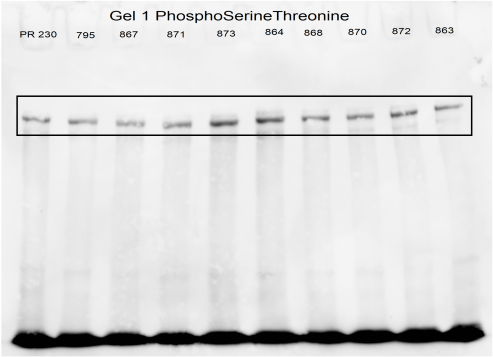

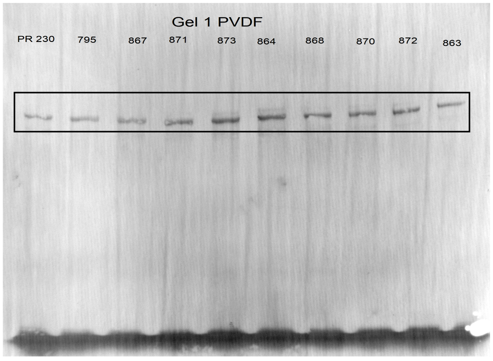

pTTN-Ser/Thr

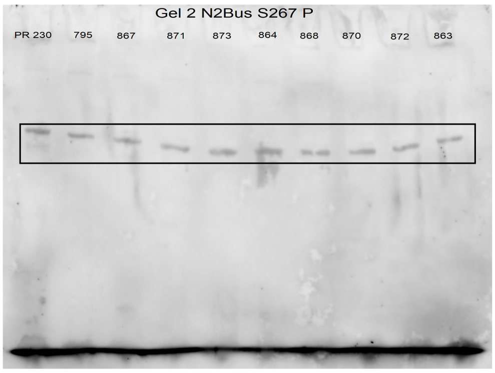

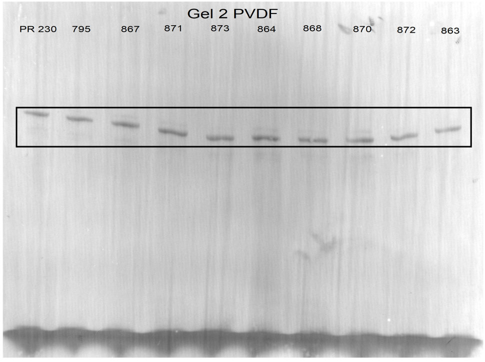

pTTN-Ser4080

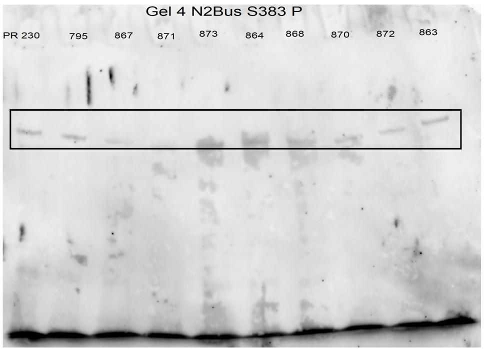

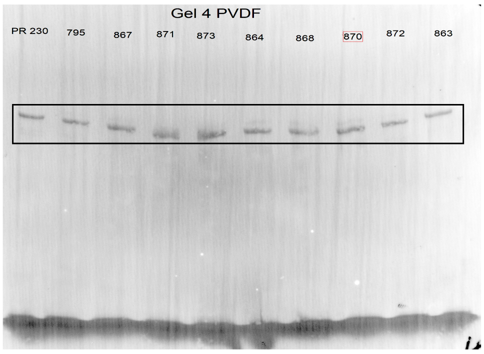

pTTN-Ser12742

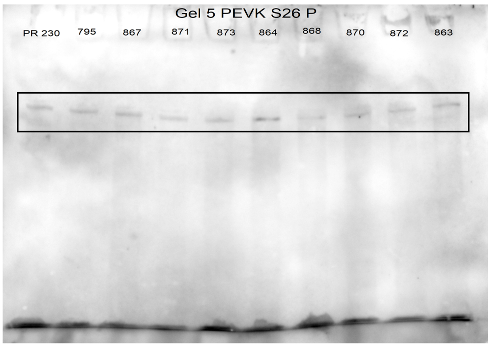

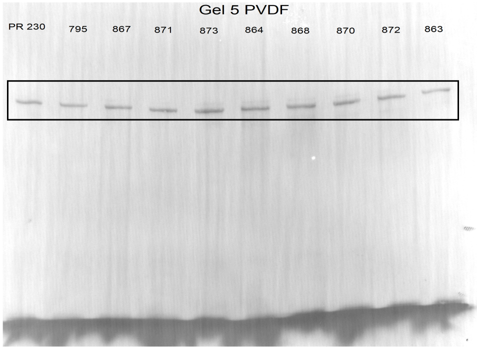

Supplement: Figure 6—figure supplement 4—source data 1. [file elife-78629-fig6-figsupp4-data1.pdf]
