# Supplementary material for: Ablation of palladin in adult heart causes dilated cardiomyopathy associated with intercalated disc abnormalities
Source: eLife. 2023 Mar 16;12:e78629. doi: 10.7554/eLife.78629 (PMC10069870; doi:10.7554/eLife.78629)
Supplement: Supplementary file 1. [file elife-78629-supp1.docx]

**Supplementary file 1.** Oligos used for quantitative real-time PCR (qRT-PCR) and clonings.

| **Primers** | **Sense** | **Reverse** |
| --- | --- | --- |
| **qRT-PCR (mouse)** |  |  |
| *Palld 200 kDa* | CATCCAGAAACTGAGGAGCC | AGCTTTCGCTGTCAGAGTCC |
| *Palld 140 kDa* | TGCTGCCTGTGCATTTTCCC | AGCTTTCGCTGTCAGAGTCC |
| *Palld 90 kDa* | AGGAGCCCTCGACACCCA | TCCTGTTCCAGGCGCACTTGG |
| *Mypn* | CATGCTTTGCTTCCAACATT | GGCTTCTGGATTCGATTCAT |
| *Nppa* | CACAGATCTGATGGATTTCAAGA | CCTCATCTTCTACCGGCATC |
| *Nppb* | GTCAGTCGTTTGGGCTGTAAC | AGACCCAGGCAGAGTCAGAA |
| *Myh6* | CGCATCAAGGAGCTCACC | CCTGCAGCCGCATTAAGT |
| *Myh7* | CGCATCAAGGAGCTCACC | CTGCAGCCGCAGTAGGTT |
| *Actc1* | gcttccgctgtccagaga | atgccagcagattccatacc |
| *Acta1* | AATGAGCGTTTCCGTTGC | ATCCCCGCAGACTCCATAC |
| *Ankrd1* | gctggagcccagattgaa | ctccacgacatgcccagt |
| *Nebl* | gcggatacacctgaaaacct | cctgcctttgctttcttcaa |
| *Ldb3* | GGAAGATGAGGCTGATGAGTGG | TGCTGACAGTGGTAGTGCTCTTTC |
| *Pdlim3* | tgggggcatagacttcaatc | gctttgcttcctggagtgat |
| *Des* | Gtgactgaagcttgtcgctgtc | Cccagctaggaagcaaggtaca |
| *Tnnc1* | GAAGGACGACAGCAAAGGGA | CGGAAGAGATCCGACAGCTC |
| *Atp2a2* | tcgaccagtcaattcttacagg | cagggacagggtcagtatgc |
| *Mrtfa* | ATGACATGAAGGTGGCAGAG | TGACTTGGTCTTGGTAGGCA |
| *Srf* | Gcttcaccagatggctgtgata | Aataagtggtgccgtcccttg |
| *Col1a2* | GTAACTTCGTGCCTAGCAACA | CCTTTGTCAGAATACTGAGCAGC |
| *Col3a1* | CCTGGCTCAAATGGCTCAC | CAGGACTGCCGTTATTCCCG |
| *Ccn2* | TGACCTGGAGGAAAACATTAAGA | AGCCCTGTATGTCTTCACACTG |
| *Acta2* | CTGACAGAGGCACCACTGAA | CATCTCCAGAGTCCAGCACA |
| *Tgfb1* | TGGAGCAACATGTGGAACTC | CAGCAGCCGGTTACCAAG |
| *Bcl2* | GTACCTGAACCGGCATCT | GGGGCCATATAGTTCCACAA |
| *Bax* | GTCAGCGGCTGCTTGTCT | GGTCCCGAAGTAGGAGAGGA |
| *Tp53* | GTACCTTATGAGCCACCCGA | CTTCTGTACGGCGGTCTCTC |
| *Egr1* | CTTCAGCCGAAGTGACCACC | GCTCTTCCGTTCCTTCTGCC |
| **qRT-PCR (human)** | | |
| *MYPN* | GAATCGAATCCAGAAGCCAA | GGGTACTGCTGGAGGAATGA |
| *PALLD 5’ (200 kDa isoform)* | Cagctgcacctcgattcat | Ctttcccttcacagaaccatct |
| *PALLD 3’ (all isoforms including the C-term)* | CAGGAGCGATTCTTCAGACC | ACCCACTGACTTTGCAGTCC |
| *ANKRD1* | GGTGAGGACTGGCCACTATG | CCTTCTCTGTCTTTGGCGTT |
| *HPRT* | GCTGAGGATTTGGAAAGGGT | GAGGGCTACAATGTGATGGC |
| **Clonings** | | |
| pGBKT7 human MYPN N-term start-Ig2 (bp 233-1798; aa. 1-522; NM_032578.3) | tttcatatg/ATGCAAGACGACAGCATA GAAGCTTCTACT | tttggatcc/GCCGTATTTGTTGCTTGCAGTACATGTGAA |
| pGBKT7 human MYPN C-term Ig3-end (bp 3044-4195; aa. 938-1320; NM_032578.3) | tttcatatg/CCCACGGGCAAGTGTATTGCTCCCATCTTT | tttggatcc/TTAAAGTTCATCACTCTCCACTACACTCCG |
| pGBKT7 human MYPN full-length (bp 233-4195; aa. 1-1320; NM_032578.3) | tttcatatg/ATGCAAGACGACAGCATAGAAGCTTCTACT | tttggatcc/TTAAAGTTCATCACTCTCCACTACACTCCG |
| pGBKT7 human PALLD full-length (bp 212-3583; aa. 1-1123; NM_001166108) | catggaggccgaattc/ATGTCAGGGACCTCCTCCCATGAG | gcaggtcgacggatcc/TTAATGTCGAGAAATGTAAACGTCCAGCCTGGCAGTACA |
| pGBKT7 human PALLD N-term start-Ig2 (bp 212-1795; aa. 1-528; NM_001166108) | catggaggccgaattc/ATGTCAGGGACCTCCTCCCATGAG | gcaggtcgacggatccta/TCCATAATCATTTCTTGCTGAACATGTAAAGATCCC |
| pGBKT7 mouse PALLD C-term Ig3-end (bp 1146-2309; aa 267-680; BC127081) | atccaagcttctcgag/AACGCAACAGCTCCCTTCTTTGAGATG | gcaggtcgacggatcc/TCACAGGTCTTCACTTTCTACCAAGCCG |
| pGADT7 human CARP full-length (bp 249-1208; aa. 1-319; NM_014391.3) | catggaggccgaattc/ATGATGGTACTGAAAGTAGAGGAACTGGTC | gcaggtcgacggatcc/TCAGAATGTAGCTATGCGAGAGGTCTTG |
| pGADT7-AD human FHOD1 (bp 3005-3607, aa. 965-1164; NM_013241.2) | catggaggccgaattc/CCGCAGGCGGCCCGTGAAG | gcaggtcgacggatcc/TCACACCTCCAGGCCAGGACC |
| pGADT7-AD human FHOD1 (bp3005-3268, aa. 965-1052; NM_013241.2) | ggaggccagtgaattc/CCGCAGGCGGCCCGTGAAG | cgagctcgatggatcccta/ACTAGCATGACTGTCAGCATCTCCCC |
| pGADT7-AD human FHOD1 (bp 3152-3607, aa. 1014-1164; NM_013241.2) | ggaggccagtgaattc/ATGATCACCGAGACAGAGAAGTTCTCAG | cgagctcgatggatcc/TCACACCTCCAGGCCAGGACC |
| pFN21A HaloTag CMV Flexi vector human PALLD full-length (bp. 212-3583, aa. 1-1123; NM_001166108.2) | gcgataacgcgatcgcc/ATGTCAGGGACCTCCTCCCATGAG | agcccgaattcgtttaaac/TTAATGTCGAGAAATGTAAACGTCCAGCCTG |
| pNLF1-N [CMV Hygro] mouse FHOD1 Y2H clone (bp3106-3711, aa. 997-1197; NM_177699.4) | ttctggcgggctcgagc/CCACAGGCAGCAAGGGATGTACG | gttgagctctgaattc/TCACACCTCTAGACCAGGAGCTTTGC |
| pNLF1-C [CMV Hygro] mouse CARP (bp 64-1023; aa. 1-319; NM_013468.3) | gatcgcttccgaattc/ATGATGGTACTGAGAGTAGAGGAGCTG | gaagacgccgctcgagcc/GAATGTAGCTATGCGAGAGTTCTTGTAGG |

Vector specific sequence is written in lowercase letters.
